# Supplementary figures and images for: Evolution of natural lifespan variation and molecular strategies of extended lifespan in yeast
Source: eLife. 2021 Nov 9;10:e64860. doi: 10.7554/eLife.64860 (PMC8612763; doi:10.7554/eLife.64860)

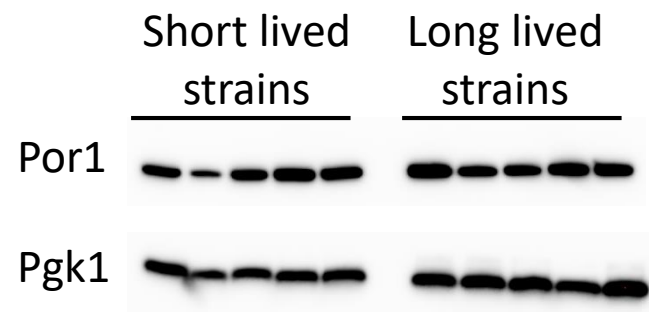

30kDA

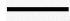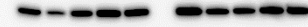

Por1

45kDA

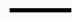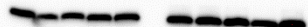

Pgk1

Supplement: Figure 6—figure supplement 4—source data 1. [file elife-64860-fig6-figsupp4-data1.zip › Kaya-BlotsImages/Kaya-BlotsImages.pdf]

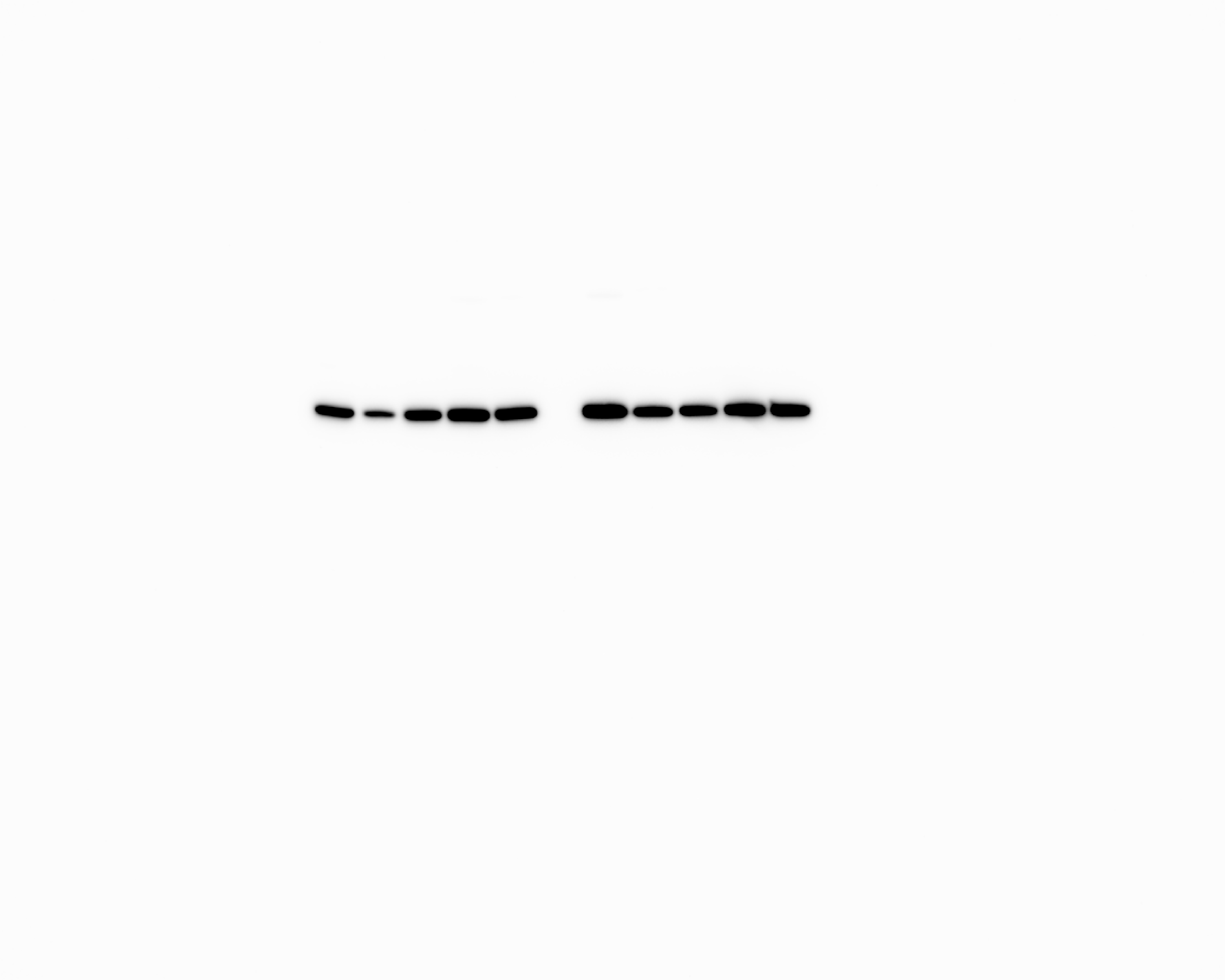

Supplement: Figure 6—figure supplement 4—source data 1. [file elife-64860-fig6-figsupp4-data1.zip › Kaya-BlotsImages/Kaya-Chemiluminescence1.tif]
