# Supplementary material for: Evolution of natural lifespan variation and molecular strategies of extended lifespan in yeast
Source: eLife. 2021 Nov 9;10:e64860. doi: 10.7554/eLife.64860 (PMC8612763; doi:10.7554/eLife.64860)
Supplement: Transparent reporting form [file elife-64860-transrepform1.docx]

***eLife’s* transparent reporting form**

We encourage authors to provide detailed information *within their submission* to facilitate the interpretation and replication of experiments. Authors can upload supporting documentation to indicate the use of appropriate reporting guidelines for health-related research (see [EQUATOR Network](http://www.equator-network.org/%20)), life science research (see the [BioSharing Information Resource](https://biosharing.org/)), or the [ARRIVE guidelines](http://www.plosbiology.org/article/info:doi/10.1371/journal.pbio.1000412) for reporting work involving animal research. Where applicable, authors should refer to any relevant reporting standards documents in this form.

If you have any questions, please consult our Journal Policies and/or contact us: [editorial@elifesciences.org](mailto:editorial@elifesciences.org).

**Sample-size estimation**

- You should state whether an appropriate sample size was computed when the study was being designed
- You should state the statistical method of sample size computation and any required assumptions
- If no explicit power analysis was used, you should describe how you decided what sample (replicate) size (number) to use

Please outline where this information can be found within the submission (e.g., sections or figure legends), or explain why this information doesn’t apply to your submission:

Not applicable. None of our experiments needs formal power analysis.

**Replicates**

- You should report how often each experiment was performed
- You should include a definition of biological versus technical replication
- The data obtained should be provided and sufficient information should be provided to indicate the number of independent biological and/or technical replicates
- If you encountered any outliers, you should describe how these were handled
- Criteria for exclusion/inclusion of data should be clearly stated
- High-throughput sequence data should be uploaded before submission, with a private link for reviewers provided (these are available from both GEO and ArrayExpress)

Please outline where this information can be found within the submission (e.g., sections or figure legends), or explain why this information doesn’t apply to your submission:

**Relative growth measurements**

We used optical density as measure of growth rate among the strains. We repeated each assay at least in three biological replicates and all the values from each replicates are provided in Supplementary File 1 as a source file.

**Replicative lifespan analysis**

We assayed RLS of these isolates at 30 °C using 2different growth conditions: YPD medium, and YPG medium. Under YPD and YPG conditions, most of the natural isolates were assayed at least in two independent RLS analysis, each with 20 mother cells. N numbers and statistical calculations are provided in Supplementary File 1 as a source file.

**RNA-sequencing**

Three independent cultures (biological replicates) for each strain were collected at the OD_600_=0.4 to isolate DNA and RNA and metabolites (below) from each culture and pellets were used for RNA-seq library construction and transcriptome analysis. Details are included in materials and methods section. Raw and normalized values of RNA-seq reads for each biological replicates are provided in Supplementary File 2 as a source file.

**Metabolite profiling**

For most of the strains we perfumed only one metabolite profiling. We had three repeats for the randomly selected three long and three short lived strains. We processed all the sample at the same time to decrease the batch effect of sample preparation and applied extensive data normalization and imputation method for data analysis as it is explained in the method section. Normalized metabolite values are provided in Supplementary file 2 as a source file.

**Statistical reporting**

- Statistical analysis methods should be described and justified
- Raw data should be presented in figures whenever informative to do so (typically when N per group is less than 10)
- For each experiment, you should identify the statistical tests used, exact values of N, definitions of center, methods of multiple test correction, and dispersion and precision measures (e.g., mean, median, SD, SEM, confidence intervals; and, for the major substantive results, a measure of effect size (e.g., Pearson's r, Cohen's d)
- Report exact p-values wherever possible alongside the summary statistics and 95% confidence intervals. These should be reported for all key questions and not only when the p-value is less than 0.05.

Please outline where this information can be found within the submission (e.g., sections or figure legends), or explain why this information doesn’t apply to your submission:

Replications are explicitly mentioned in the material method section and raw and normalized values for each replicates provided as supplementary files.

(For large datasets, or papers with a very large number of statistical tests, you may upload a single table file with tests, Ns, etc., with reference to sections in the manuscript.)

**Group allocation**

- Indicate how samples were allocated into experimental groups (in the case of clinical studies, please specify allocation to treatment method); if randomization was used, please also state if restricted randomization was applied
- Indicate if masking was used during group allocation, data collection and/or data analysis

Please outline where this information can be found within the submission (e.g., sections or figure legends), or explain why this information doesn’t apply to your submission:

Regression methods are thoroughly described in the materials and methods.

For clustering, pearson's correlation was used. We extensively explained statistical methods and approaches that were used for data analysis. All regression results with adjusted p values are provided in Supplementary File 2 as source file.

**Additional data files (“source data”)**

- We encourage you to upload relevant additional data files, such as numerical data that are represented as a graph in a figure, or as a summary table
- Where provided, these should be in the most useful format, and they can be uploaded as “Source data” files linked to a main figure or table
- Include model definition files including the full list of parameters used
- Include code used for data analysis (e.g., R, MatLab)
- Avoid stating that data files are “available upon request”

Please indicate the figures or tables for which source data files have been provided:

This is clearly delineated in figures, method sections and supplemental data files that source files for figure 1,2,3,4,5, 7,8 were provided in the related supplementary files.
